# Human Physiology

**Published:** 1851-01

**Authors:** 


*Human Physiology.* By Robley Dunglison, M. D., etc., etc., etc. With nearly five hundred illustrations. Seventh Edition. Thoroughly revised and extensively modified and enlarged. In two volumes. Philadelphia: Lea & Blanchard. 1850.

It is now more than fifteen years since the publication of the first edition of "Dunglison's Physiology." During this lapse of time the work has passed through several editions, each of which has received, from the hands of the Author, revision, together with such additions as were required by the constant developments and changes incident to the progress of physiological knowledge. From the first appearance of the work, it has been highly prized by medical pupils, and by practitioners of medicine, and in most, if not all, the medical institutions of the country, it has been recognized as a text book in the department of medical education of which it treats. It has also been received with great favor on the other side of the Atlantic. So well known and established, indeed, is its character, that commendation on the part of the Journalist at this period in its history, would not only be supererogatory, but be deemed by the reader officious almost to the degree of impertinence.

In noticing the publication of the *seventh* edition the readiest and best method of doing justice to our readers, and to the author, will be to make some quotations from the preface. The author therein states that he has bestowed more care on this, than any previous review of the work; that the present edition has been "subjected to an entire scrutiny, not only as regards the important matters of which it treats, but the language in which they are conveyed." * * * * * * *

" On the whole subject of physiology proper, as it applies to the functions executed by the different organs, the present edition, the author flatters himself, will be found to contain the views of the most distinguished physiologist of all periods. The contributions to the science of life, of late years, have been rich and varied; and to collect and weigh them, and to separate the most worthy and valued, has been a work of no little discriminating labor—but to the author a labor of love, inasmuch as they are subjects which he has been long accustomed to investigate, and on which he has annnally to treat before the class of Institutes of Medicine of the Jefferson Medical College."

* * " On no former occasion, has the author felt as satisfied with his endeavors to have the work on a level with existing state of the Science."

For sale by Phinney & Co., and Derby & Co.

